# Tranexamic acid in laparoscopic sleeve gastrectomy: bleeding control amid thrombotic concerns and hemodynamic instability: a randomized double-blinded clinical trial

**DOI:** 10.1590/acb416226

**Published:** 2026-10-19

**Authors:** Osman Anıl Savaş, Selçuk Mercan, Amir Mahdi Akbari, Mehrdad Sheikhvatan

**Affiliations:** 1 Okan University – Faculty of Medicine – Department of General Surgery – Istanbul – Turkey.

**Keywords:** Tranexamic Acid, Gastrectomy, Safety, Hemorrhage

## Abstract

**Purpose:**

Laparoscopic sleeve gastrectomy (LSG) carries a risk of intraoperative and postoperative bleeding. Tranexamic acid (TXA) has been proposed to reduce blood loss. We evaluated the efficacy of TXA in preventing perioperative bleeding in patients undergoing LSG.

**Methods:**

In a randomized clinical trial, 185 morbidly obese patients scheduled for elective LSG were randomly assigned to receive TXA (n = 93) or a placebo (n = 92) before surgery. The rate of bleeding requiring blood transfusion, 24-hour serum hemoglobin levels, postoperative hemodynamic indices, and the incidence of postoperative thrombotic events were assessed.

**Results:**

Intraoperative bleeding occurred less frequently in the TXA group than in the placebo group (1 *versus* 5.5%, *p* = 0.012). Postoperative hemoglobin levels were higher in the TXA group (12.5 ± 1.2 g/dL *versus* 11.1 ± 1.5 g/dL, *p* = 0.002), while transfusion requirements, hemodynamic stability, and length of hospital stay were similar between groups. No postoperative thromboembolic events were observed. Multivariable analysis confirmed TXA as an independent predictor of reduced bleeding and higher postoperative hemoglobin levels.

**Conclusion:**

TXA significantly reduced intraoperative bleeding and improved postoperative hemoglobin levels in LSG without increasing hemodynamic instability or the risk of thromboembolic events.

## Introduction

Obesity has become a leading global health concern, with steadily rising prevalence in recent decades and significantly contributing to morbidity and mortality and overall healthcare expenditure^1^. Bariatric surgery continues to be the most effective long-term treatment for morbid obesity, and expected weight loss is maintained over time and leads to partial or complete resolution of related comorbid conditions like type 2 diabetes mellitus, hypertension, and obstructive sleep apnea^2,3^. Currently, laparoscopic sleeve gastrectomy (LSG) has gained widespread acceptance due to its relative technical simplicity, much promising metabolic response, and lower complication rates among all other procedures^4,5^.

Despite the generally favorable safety profile, postoperative hemorrhage continues to be one of the most clinically significant complications of sleeve gastrectomy^6^. Hemorrhage can occur intraoperatively or in the early postoperative period and most commonly emanates from the gastric staple line or short gastric vessels^7^. Bleeding following LSG can result in hemodynamic instability, prolonged hospitalization, increased blood product transfusion, reoperation, and, in extreme circumstances, mortality^8^. Consequently, strategies aimed at minimizing perioperative blood loss are important in improving surgical outcomes and patient safety.

Tranexamic acid (TXA) is a synthetic lysine competitor that has antifibrinolytic properties due to its ability to competitively inhibit the conversion of plasminogen to plasmin^9^. TXA has been explored and is effective in many areas of surgery, including orthopedic, cardiac, trauma, and general surgery^10,11^. TXA has been extensively evaluated in randomized controlled trials and meta-analyses that prove it can reduce intra- and post-operative blood loss and transfusions with no increase in thromboembolic events^12-14^.

There has been a growing interest in the use of TXA in the context of bariatric surgery, especially in procedures conducted with large staple lines such as sleeve gastrectomy^15^. Nonetheless, the patient who undergoes bariatric surgery normally has a prothrombotic condition associated with obesity. Therefore, the application of TXA in sleeve gastrectomy has been associated with mixed findings regarding both efficacy and safety.

In view of the clinical interest of postoperative bleeding regarding LSG and the possible advantages offered by TXA, it seems appropriate to focus additional attention upon its use in this situation. The aim of this research was to determine the efficacy of TXA in preventing bleeding during the perioperative period amongst patients who have had LSG, as well as its effect upon transfusion needs and safety.

## Methods

### Study design

This study was conducted after obtaining ethical approval from the institutional ethical committee (Ethical Code: E-10840098-202.3.02-1010). The trial was registered at ClinicalTrials.gov (NCT06038981) on 8 of September of 2023. Written informed consent was obtained from all patients before participation. The study was in accordance with the guidelines of the Consolidated Standards of Reporting Trials (CONSORT) (Fig. 1).

**Figure 1:**
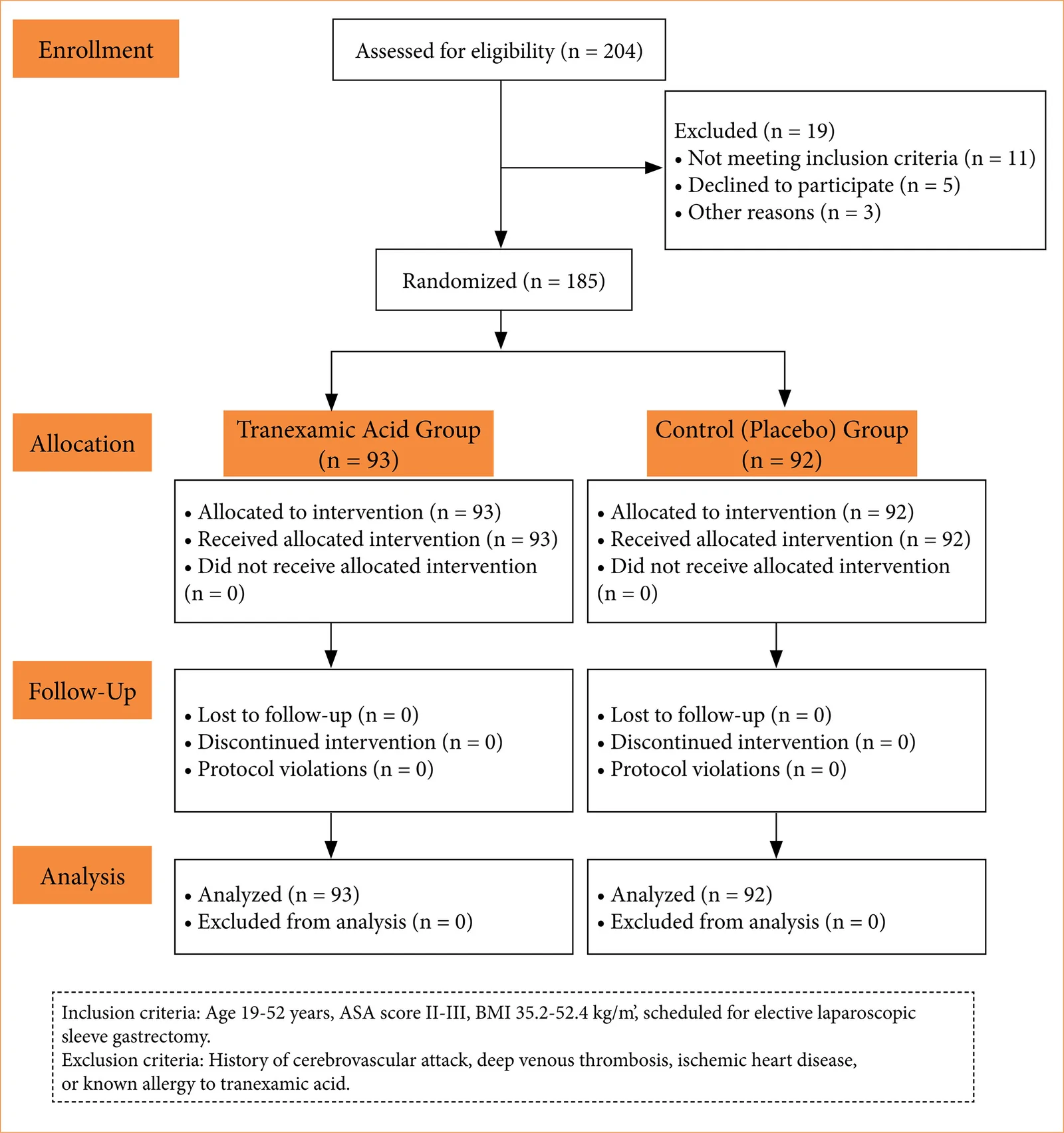
The Consolidated Standards of Reporting Trials (CONSORT) flow diagram describing how to select the study participants.

BMI: body mass index. Source: Elaborated by the authors.

Prior to study initiation, a prospective sample size calculation was performed based on the anticipated reduction in perioperative blood loss following TXA administration based on a previous study^11^. Assuming a moderate effect size of 0.45, a two-sided α error of 0.05, and a statistical power of 80%, the minimum required sample size was calculated to be 82 patients per group. Considering a potential dropout rate of approximately 10%, an enrollment target of 185 patients was determined to ensure adequate statistical power for the primary outcome analysis. A total of 204 patients were assessed for eligibility between January and December 2024. Nineteen patients were excluded, and 185 patients were randomized into the TXA group (n=93) or placebo group (n = 92) (Fig. 1).

Randomization was performed using a computer-generated random allocation sequence with a 1:1 allocation ratio. Block randomization with a fixed block size of four was applied to maintain balanced group assignment throughout the study period. No stratification was used. Allocation concealment was ensured using sequentially numbered, opaque, sealed envelopes prepared by an independent investigator who was not involved in patient recruitment, intervention administration, or outcome assessment.

The patients aged between 19 and 52 years old, with ASA scores II or III and initial body mass index (BMI) ranged from 35.2 to 52.4 kg/m^2^. Patients with a history of cerebrovascular attack, deep venous thrombosis, ischemic heart disease, and known allergy to TXA were excluded from the study. The participants were randomly assigned to preoperatively receive TXA (1 g/50 mL, intravenously, as a single dose, n = 93) or placebo (0.9% normal saline/50 mL, intravenously, n = 92). Double blinding was implemented by ensuring that both the participants and the clinical investigators, including surgeons, anesthesiologists, nursing staff, and outcome assessors, were unaware of treatment allocation throughout the study.

TXA and placebo solutions were prepared by an independent pharmacist who was not involved in patient management or data collection. Both study solutions were identical in volume (50 mL), appearance, and method of intravenous administration. The prepared syringes/infusion bags were labeled with anonymized study codes to maintain concealment until completion of the final statistical analysis. The patients were matched in both groups according to the factors that may be effective in reducing bleeding, including surgical technique, the surgical team (including surgeon and surgeon’s assistance), and comorbidities. Both physicians and patients were blinded to the type of treatment. All surgeries were performed by one surgical team with the same technique.

Baseline information including demographics, BMI, history of previous surgery, duration of surgery, and laboratory parameters were all extracted from the hospital recorded files. Also, operative data with respect to intraoperative bleeding, requiring blood transfusion, and the value of 24-hour serum hemoglobin level were collected by the patients’ following-up within hospitalization. The patients were also assessed within hospital stay about thrombotic events, including deep vein thrombosis or pulmonary emboli.

### Surgical technique

LSG was performed using the standard 5-trocar technique by the same team. Intra-abdominal pressure was maintained between 12-15 mmHg. LigaSure was used for the dissection of the gastroepiploic omentum and adhesions. The stapler line was either clipped or sutured according to the surgeon’s preference. Resection began 2–4 cm from the pylorus. The gastroesophageal junction was approached without getting closer than 1 cm. Resection was performed over a 36 F bougie. Covidien brand staplers appropriate for the thickness of the tissue were used in gastric transection. The stapler line was either clipped or sutured repeatedly according to the surgeon’s preference. A leak test with methylene blue was performed for each patient intraoperatively. A Jackson Pratt drain extending to the stapler line was placed. Trocar entry sites were sutured with 3/0 vicryl, and subcutaneous bupivacaine was applied to incision sites before the patient was awakened.

Patients were mobilized six hours after surgery, and respiratory physiotherapy was administered. Twelve hours postoperatively, all patients without signs of bleeding received a prophylactic dose of low-molecular-weight heparin. An oral methylene blue test was conducted on the second postoperative day, and a liquid diet was initiated. Patients were discharged on the third day.

### Study outcomes

The primary outcome of the study was the bleeding control-related parameters, including intraoperative bleeding, transfusion units required, and postoperative serum hemoglobin level. The secondary outcome included postoperative blood pressure change, duration of hospital stay, total operation time, and thromboembolic events including deep vein thrombosis or pulmonary emboli. Intraoperative bleeding was defined as any bleeding occurring during laparoscopic sleeve gastrectomy that requires intraoperative hemostatic intervention beyond routine dissection.

### Statistical analysis

Descriptive statistics were used to summarize baseline characteristics. Continuous variables were reported as means ± standard deviations or medians with interquartile ranges, and categorical variables as counts and percentages. Comparisons between the two groups were performed using Student’s t-test or Mann–Whitney’s U test for continuous variables, and χ^2^ or Fisher’s exact test for categorical variables. Univariate and multivariable logistic regression models were used to assess the difference in surgical outcome between the groups, adjusting for potential confounders including baseline parameters. *Odds ratios* (ORs) with 95% confidence intervals (95%CI) were reported. Model calibration was assessed using the Hosmer–Lemeshow’s test. Statistical significance was set at *p* < 0.05. Analyses were performed using Statistical Package for the Social Sciences (version 18, SPSS Inc., Chicago, IL, United States of America).

## Results

A total of 185 patients were included in the study receiving perioperative TXA or normal saline as the placebo. There were no significant differences between the two groups in terms of baseline clinical characteristics including mean age, mean BMI, gender, history of comorbidities (diabetes mellitus and systemic arterial hypertension), medication use (antihypertensive and hypoglycemic drugs), and previous abdominal surgeries (Table 1). Additionally, the surgical durations were similar in the two groups. There was no difference in baseline hemodynamic variables including heart rate, blood pressure, respiratory rate, and arterial oxygen saturation either. The mean operation time in TXA and placebo groups was 48.4 ± 2.6 min and 43.6 ± 1.9 min, respectively, with no difference (*p* = 0.224).

**Table 1:** Baseline characteristics of study population.

Item	Tranexamic acid group (n = 93)	Control group (n = 92)	*p* -value
**Gender, %**			0.126
Male	9 (9.7)	16 (17.4)	
Female	84 (90.3)	76 (82.6)	
Mean age, year	34.7 ± 3.75	33.8 ± 3.14	0.961
Mean weight, kg	116.2 ± 10.6	112.8 ± 9.12	0.458
Mean height, cm	165.6 ± 4.4	168.2 ± 5.4	0.712
Mean body mass index, kg/m^2^	41.1 ± 2.8	40.2 ± 2.5	0.246
History of hypertension	19 (20.4)	17 (18.5)	0.746
History of diabetes	6 (6.4)	4 (4.3)	0.342
History of hyperlipidemia	12 (12.9)	9 (9.7)	0.740
Current smoking	6 (6.4)	7 (7.6)	0.821
Using antihypertensive drug	17 (18.2)	15 (16.3)	0.665
Using hypoglycemic drug	6 (6.4)	3 (3.2)	0.229
**Laboratory indices**			
Serum hemoglobin, gr/dL	11.8 ± 1.6	11.4 ± 1.8	0.856
White blood count, /mm^3^	9,600.4 ± 982.3	10,700.6 ± 1,012.2	0.224
Platelet count, /mm^3^	386.4 ± 22.7	354.7 ± 20.6	0.621
Fasting blood sugar, mg/dL	96.6 ± 4.5	94.2 ± 3.9	0.126
Fasting high-density lipoprotein, mg/dL	36.4 ± 2.3	38.8 ± 2.9	0.256
Fasting low-density lipoprotein, mg/dL	99.7 ± 12.1	97.5 ± 10.6	0.244
Serum creatinine, mg/dL	0.99 ± 0.02	0.98 ± 0.04	0.723

Source: Elaborated by the authors.

Regarding operative outcome, 5.5% of patients in the placebo group suffered from intraoperative bleeding, while the bleeding rate reduced to 1% in TXA group indicating a significant difference (*p* = 0.012). None of the patients with bleeding required additional operations. Moreover, the mean postoperative value of serum hemoglobin was significantly higher in the TXA group (12.5 ± 1.2 g/dL *versus* 11.1 ± 1.5 g/dL, *p* = 0.002). However, no difference was revealed in the transfusion units required between the two groups (1.2 units/patient *versus* 1.8 unit/patient, *p* = 0.456). Blood pressure dropped below 100/60 mmHg in none of the patients, and orthostatic hypotension developed only in two patients—one in the TXA group and another in control group. With blood transfusion and close clinical monitoring, bleeding stopped spontaneously in all studied ones. The mean hospital stay in TXA group and control group was 2.2 ± 0.6 days and 2.4 ± 0.7 days with no difference (*p* = 0.756). No thromboembolic events including deep vein thrombosis or pulmonary emboli was reported after surgery.

In multivariable linear regression model (Table 2), the use of TXA could effectively predict lower intraoperative bleeding rate (Beta = 1.494, standard error = 1.126, *p* = 0.026). In a similar model (Table 3), using TXA led to higher serum hemoglobin level postoperatively (Beta = 0.448, standard error = 0.127, *p* = 0.038).

**Table 2:** Multivariable linear regression analysis for determining the difference in bleeding rate between tranexamic acid and placebo groups.

Variable	Unstandardized coefficients		*p* -value	95% confidence interval for beta	
	Beta	Standard error		Lower bound	Upper bound
Tranexamic acid use	1.494	1.126	0.026	1.347	1.641
Female gender	-1.865	1.293	0.153	-4.433	0.703
Age	-2.793	1.089	0.002	-3.705	-1.881
Body mass index	0.651	0.619	0.296	-0.578	1.880
History of hypertension	-0.196	0.738	0.792	-1.661	1.269
History of diabetes	-1.481	0.731	0.046	-2.933	-0.029
History of hyperlipidemia	-0.085	0.296	0.775	-0.674	0.504
Current smoking	0.165	0.305	0.589	-0.440	0.770
Serum hemoglobin	-0.198	0.419	0.637	-1.030	0.634
White blood count	-1.571	1.044	0.874	-0.477	0.334
Platelet count	-3.864	2.926	0.275	-2.274	2.546
Fasting blood sugar	2.000	1.153	0.012	-1.384	6.383
Fasting high-density lipoprotein	-3.859	4.695	0.036	-12.589	5.872
Serum creatinine	-0.129	0.126	0.224	-0.239	0.124

R square = 0.310; adjusted R square = 0.223. Source: Elaborated by the authors.

**Table 3:** Multivariable linear regression analysis for determining the difference in serum hemoglobin level between tranexamic acid and placebo groups.

Variable	Unstandardized coefficients		*p* -value	95% confidence interval for beta	
	Beta	Standard error		Lower bound	Upper bound
Tranexamic acid use	0.448	0.127	0.038	0.224	0.750
Female gender	2.725	0.273	0.064	-0.745	6.194
Age	-0.148	0.037	0.157	-0.619	0.323
Body mass index	0.043	0.025	0.335	-0.275	0.362
History of hypertension	1.147	0.185	0.102	-1.204	3.497
History of diabetes	-0.294	0.053	0.113	-0.965	0.377
History of hyperlipidemia	0.363	0.039	0.068	-0.132	0.858
Current smoking	0.143	0.021	0.092	-0.122	0.409
Serum hemoglobin	0.075	0.026	0.215	-0.260	0.410
White blood count	0.120	0.012	0.065	-0.035	0.275
Platelet count	-0.766	0.151	0.124	-2.688	1.157
Fasting blood sugar,	-0.211	0.035	0.105	-0.657	0.234
Fasting high-density lipoprotein	0.112	0.019	0.109	-0.134	0.357
Serum creatinine	0.057	0.054	0.372	-0.089	0.226

R square = 0.399; adjusted R square = 0.217. Source: Elaborated by the authors.

## Discussion

Bleeding complications after the procedure have long been a very significant issue post-LSG, and it has been associated with morbidity, longer hospital stay, blood transfusion therapy, reoperation, and increase in healthcare costs^8^. Due to the long staple line across the stomach and the common occurrence of bleeding disorders that are commonly associated with morbid obesity, the use of techniques that aim to decrease the risk of bleeding during the procedure has become very important^16^. This research study helped confirm the beneficial use of TXA for the prevention of bleeding during LSG. Our study showed that the use of TXA before surgery could result in lowering intraoperative bleeding rate, as well as higher postoperative serum hemoglobin level. However, this regimen might not affect the rate of blood transfusion. Also, administrating TXA did not led to fluctuate postoperative hemodynamic status or thrombotic events within hospitalization.

TXA works as a synthetic analog of lysine and binds to the lysine-binding pockets of plasminogen in a reversible manner to inhibit its conversion to plasmin and thus prevents degradation of the fibrin clot^17^. In the context of bariatric surgery, in which extensive tissue handling and stapling lead to activation of fibrinolysis, TXA has sound biologic mechanisms in preventing and treating fibrinolysis-related hemorrhages^18^. Lastly, in terms of the observed reduction in intraoperative blood loss and hemorrhages, this study is consistent within the established biologic and physiologic properties of TXA and its proven efficacy in other surgical specialties such as orthopedic surgery, cardiovascular surgery, trauma surgery, and obstetric surgery.

Our findings agree with several previous reports investigating TXA use in bariatric procedures. Prior studies have demonstrated reduced drain output, lower postoperative hemoglobin drop, and decreased need for blood transfusion in patients receiving TXA during LSG^19-21^. Importantly, some authors have also reported a reduced incidence of reoperation due to staple-line bleeding, a complication that, although infrequent, carries significant clinical and economic consequences^22^. The present study adds to this growing body of evidence by reinforcing the effectiveness of TXA specifically in LSG, a procedure characterized by a longer staple line compared with other bariatric operations.

However, one of the major controversies in the perioperative use of TXA in bariatric surgery has been the perceived risk of thromboembolic complications due to the hypercoagulable state that exists in obese individuals. In our investigation, the use of TXA did not result in an elevated incidence of deep vein thrombosis, pulmonary embolism, and other thrombotic complications. These findings are supported by large meta-analyses that have been published in the literature and included both non-bariatric and bariatric surgery groups, who found that TXA does not confer an elevated incidence of thromboembolism if carefully used in controlled amounts and in addition to conventional thromboembolism prevention practices^23^.

Another important clinical application of TXA administration is its possible effect on the efficiency of operation. A reduction in bleeding during operation may enhance visualization of the surgical site, aid in accurate stapling, and reduce the duration of operation. In addition, a decrease in bleeding after operation may prevent the need for a surgical drain, transfusion of blood, and intensive care of patients^24^. These, in turn, may reduce hospitalization time and healthcare costs. Though these secondary outcomes may not have been considered as goals of this clinical study, these are significant advantages favoring the increased use of TXA in bariatric procedures.

Although the rate of perioperative bleeding was significantly lower in the TXA group, transfusion requirements did not differ between the study arms. This finding may be explained by the overall low incidence of clinically significant hemorrhage in elective LSG, in which most bleeding episodes are minor and can be managed conservatively without transfusion. In addition, modern perioperative blood management strategies, restrictive transfusion thresholds, and early hemostatic interventions may have further reduced the need for blood product administration in both groups. Similar observations have been reported in recent bariatric surgery literature, where TXA reduced bleeding-related parameters without demonstrating a parallel reduction in transfusion rates, likely due to the rarity of severe hemorrhagic events and limited statistical power for this outcome^24^.

Not withstanding the many advantages of the current study, there are several limitations that must be recognized. If the study is single centered, there may be potential bias and lack of generalizability. Further, there may also be the effects of varying TXA dosage strategies and the time of administration and the type of surgeries performed. There may also be an inaccuracy in the reporting of thromboembolic events in smaller studies or those with shorter periods of follow-up. Additionally, thromboembolic surveillance was limited to the in-hospital period (approximately 72 hours), which represents an important limitation of this study.

Given the substantial proportion of postoperative venous thromboembolic events occur after discharge, the reported absence or low incidence of thromboembolic complications in this cohort should be interpreted with caution. Therefore, our safety conclusions regarding thromboembolic risk are necessarily limited to the early postoperative phase and cannot exclude later events occurring after hospital discharge. Future investigations may include multicenter randomized controlled studies with more specified strategies and longer periods of follow-up.

## Conclusion

Based on this study, it appears that TXA is an efficient and safe hemostatic adjuvant in the management of perioperative bleeding associated with LSG. TXA administration is associated with an improvement in hemostatic control without an increase in thromboembolic events. Incorporating TXA in the ongoing perioperative management guidelines can potentially strengthen the safety index and result in the optimization of patient outcomes in LSG procedures.

## Data Availability

The data will be available upon request.
